# Data-driven design guide for vibrotactile display layouts by continuous mapping

**DOI:** 10.1038/s41598-025-25416-3

**Published:** 2025-11-12

**Authors:** Max vom Stein, Maximilian Hoppe, Niclas Rieger, Kai-Dietrich Wolf

**Affiliations:** 1 Chair of Mechatronics, University of Wuppertal, Wuppertal, Germany; 2 Institute for Security Systems, University of Wuppertal, Wuppertal, Germany; 3 Institut de Ciències del Mar (ICM) - CSIC, Barcelona, Spain; 4 European Centre for Medium-Range Weather Forecasts (ECMWF), Bonn, Germany

**Keywords:** Sensory processing, Neurophysiology, Biomedical engineering, Health care

## Abstract

Tactile displays are emerging as vital components across fields such as medical technology, assistive technology, virtual reality, infotech and gaming, yet their design remains hampered by an inadequate psychophysical foundation. In this work, we critically assess current tactile display research, uncovering gaps in spatial acuity data, and introduce robust, data-driven layout guidelines for vibrotactile displays (VTDs). We collected high‐resolution vibrotactile data from 33 participants across five large‐area body sites using a novel, fully automated experimental framework that employs Bayesian adaptive parameter estimation to generate continuous psychometric functions. This approach allows us to derive thresholds at any recognition rate, thereby overcoming the limitations of traditional, discrete measures. Our findings reveal that existing datasets are scattered and inconsistent, demonstrate a pronounced horizontal anisotropy especially near the body midline and expose a marked sensitivity gradient along the lower back. These insights provide a validated psychophysical basis for VTD development, paving the way for more reliable, user‐centric designs in next‐generation tactile interfaces.

**Supplementary Information:**

The online version contains supplementary material available at 10.1038/s41598-025-25416-3.

## Introduction

Tactile interfaces have attracted considerable attention in recent years, spurred by growing interest from various scientific fields and industry^1,2^. By supplementing, complementing, or even substituting other sensory modalities, they offer additional channels for information transfer, ultimately broadening our repertoire of human–machine communication strategies^2^. In surgical contexts, they enhance precision and safety^3,4^, while prosthetic users benefit from improved grasp consistency and object recognition^5,6^. Vibrotactile cues further support sensory substitution, enabling navigation^7^ and even translating auditory signals into tactile patterns^8^. In virtual reality (VR), tactile feedback boosts training effectiveness and rehabilitation outcomes^9–11^. Meanwhile, wearable devices for the infotech sector provide rapid, hands-free communication^12–15^. When multiple tactors (tactile actuators) are arranged into more complex configurations, they form tactile displays, typically designed for large-area body sites and classified into three main modalities: static (constant pressure), vibrotactile (vibrations), and electrotactile (electrical pulses). Due to the versatility, compact form factor, simplicity, and cost-effectiveness of vibration motors, vibrotactile displays (VTD) are the most prevalent^7,8,12,13,16–21^.

Despite the skin’s inherent limitations in distinguishing closely spaced stimuli, notable VTDs still rely on either no sensory metric or static two-point discrimination (S-2PD) thresholds^22–24^, which are biased^25^ and physiologically inapplicable to vibrotactile contexts^26,27^. Several state-of-the-art designs and layouts - including the battery free haptic VR platform by Yu et al.^16^, the flexible hex-grid VTD by Jung et al.^18^, the hex-grid bistable transducer VTD by Flavin et al.^21^ or the interleaving layout intended to merge adjacent tactors perceptually by Martinez et al.^19^ - are based on mechanical factors^16^ or undershot S-2PD benchmarks^18,19,21^. Yet, without robust, quantitative psychophysical limits tailored to vibrotactile stimulation, such undershooting can lead to inefficient or suboptimal designs.

Although data on vibrotactile spatial acuity are abundant, experimental designs differ considerably. We classified studies into two most common task paradigms for deriving engineering-grade benchmarks which are vibrotactile 2PD (VT-2PD)^28–30^ and vibrotactile relative direction discrimination (VT-RDD)^31–34^. In the VT-2PD paradigm, a stimulus is presented either from one or from two tactors simultaneously at fixed separations, and participants report whether they perceive one or two distinct points. While some studies interpret these thresholds as the minimum spacing necessary for pattern resolution^28,29^, others suggest they represent the maximum spacing to elicit phantom sensations^30^. In VT-RDD, an initial stimulus is followed by a second from an adjacent tactor, with participants identifying its direction in a two-alternative forced-choice (2AFC, left/right or above/below) or 3AFC paradigm, where the central tactor may be reactivated. Variability in experimental design and testing parameters further complicates direct comparison and practical application in VTD layouts.

To address these challenges, we introduce a novel, data-driven method for designing VTDs that overcomes the limitations of static and discrete threshold measures. Our approach refines the VT-RDD paradigm by incorporating a two-point contact element from VT‐2PD and employing Bayesian adaptive parameter estimation to generate continuous psychometric functions^25,26,35^. While psychometric function fitting with adaptive procedures is well established in psychophysics, to the best of our knowledge it has not previously been applied to vibrotactile spatial acuity studies. Our method enables the derivation of significant thresholds at any recognition rate (RR), tailored to specific design requirements, and distinguishes robust from labile perceptual regions through psychometric function slopes. Moreover, our fully automated apparatus offers quasi-continuous adjustable inter-stimulus distances with a technical resolution of 0.1 mm and practical measurements down to 2.5 mm center-to-center, eliminating experimenter bias and minimizing interpolation error.

In summary, our study makes three key contributions. First, we introduce a robust, high-resolution dataset of vibrotactile spatial acuity - derived from 33 participants across five body sites in two measuring directions using continuous, Bayesian adaptive measurements - that transcends previously employed single-threshold approaches. Second, we reveal marked directional anisotropy, notably a pronounced horizontal advantage near the body midline alongside regional variability that occurs mainly in the horizontal direction at the lower back, where it manifests as a sensitivity gradient. Third, by leveraging these continuous psychometric functions, we derive statistically validated thresholds and propose practical, orientation-specific design guidelines for next-generation vibrotactile displays.

## Results and discussion

### A robust dataset for vibrotactile display design

**Fig. 1:**
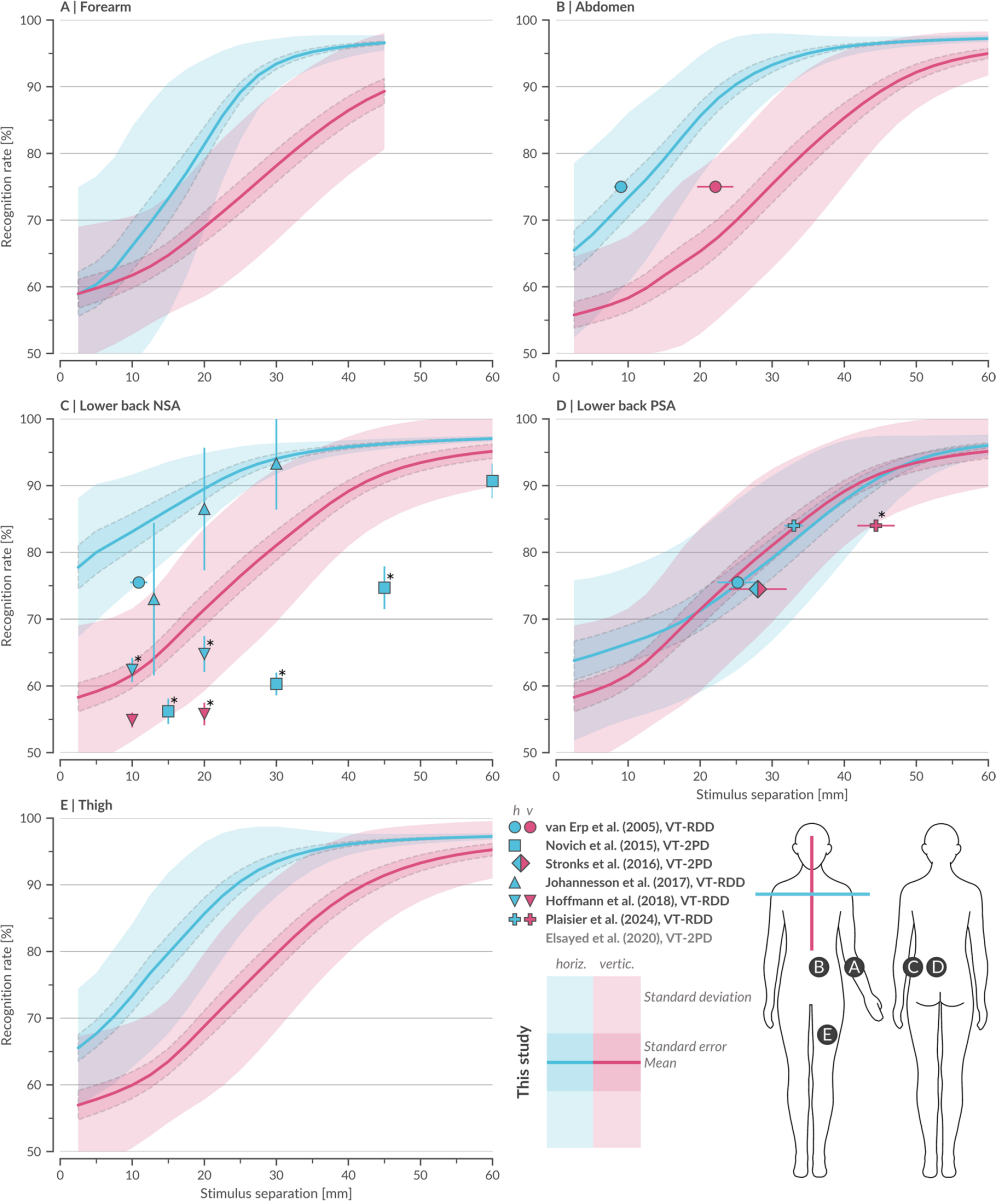
Mean psychometric functions of vibrotactile spatial acuity across body sites alongside vibrotactile data from reference literature. Mean psychometric functions (MPF) from this study (*N* = 33) showing recognition rate as a function of stimulus separation (StS) for five body sites (BS, A–E), measured in both horizontal (MPF_BS, h_ blue) and vertical (MPF_BS, v_ red) orientations, with ± 1 standard error and ± 1 standard deviation. Forearm measurements were limited to 45 mm due to space constraints. Vibrotactile data points from seven reference studies^28–34^ were normalized by the theoretical guess rate (Methods 4.6.1, raw data at Zenodo: ref_data_vt) and are presented with ± 1 standard error as x- or y-errors, color-coded for horizontal and vertical thresholds. Reference data points were tested for significant difference from our data using Bonferroni-corrected z-tests or, when z-tests were not possible, t-tests (marked with *, Methods 4.5). Due to measurement placement, peripheral-spine area (PSA) and near-spine area (NSA) share the same vertical data. Reference studies conducted their back measurements at different heights (see Fig. 6), but no statistically significant effect of height was observed. Data from Elsayed et al.^30^ out of bounds. Stronks et al.^29^ provided diagonal data interpreted as blend of horizontal and vertical. Detailed statistical analyses provided on Zenodo (statistic_data_fig1).

We gathered high-resolution vibrotactile data from 33 participants across five large-area body sites (forearm, abdomen, lower back near-spine area (NSA), lower back peripheral-spine area (PSA), and thigh), with measurement obtained in both horizontal and vertical orientations (Fig. 1). Using our fully automated apparatus, we employed Bayesian adaptive parameter estimation to sample stimulus separations continuously and maximize information gain per trial^25,26,35^. This approach yielded psychometric functions for each participant, body site, and orientation, providing RR over an entire range of stimulus separations (StS). In contrast to studies restricted to one or two predefined thresholds^29–31,34^, our dataset thus supports flexible design decisions: designers can select any RR (e.g., 75%, 85%, 95%) and directly infer the corresponding tactor spacing threshold. The obtained participant-averaged mean psychometric functions (MPF) and exhibit a clear sigmoidal trend, underscoring the robustness of our dataset and capturing both low- and high-acuity ranges.

Next, we evaluated our results alongside seven key studies that directly assessed vibrotactile spatial acuity for body sites where literature data were available, including lower back and abdomen (Fig. 1B, more data for other body sites out of bounds). Despite normalization to a common theoretical guess rate (TGR) - reflecting the probability of correct responses under unbiased random guessing (see Methods 4.6.1) - these published data remain scattered. For example, in the NSA of the lower back, the maximal spread in reported RR exceeds 30% (Fig. 1C), indicating unresolved biases potentially due to differences in experimental design, testing parameters, and response paradigms.

We further tested whether our results aligned with reported RR depending on the measuring approach (i.e. VT-RDD versus VT-2PD) using Bonferroni-corrected z-tests and t-tests (see Methods 4.5). Studies employing VT-RDD converged most closely with our dataset, with similarity (see Methods 4.5) found in 69% (*n* = 13) of reported values, whereas VT-2PD tended to produce lower thresholds, aligning in only 11% (*n* = 19) of reported values. Moreover, consistent with the documented instability of S-2PD^24,25^, VT-2PD demonstrated high variability across studies, suggesting an influence of uncontrolled test parameters.

Similarly, we assessed whether the TGR - and, by extension, the experimental design - impacted the similarity to our data. We found that 86% of reported values with a TGR similar to ours (TGR=½, *n* = 7) aligned with our recorded RRs. However, similarity declined as TGR deviation increased: agreement with our data was 57% with a TGR of ⅓ (*n* = 7) and only 7% with a TGR of 0 (*n* = 14). Additional factors likely include tactor type, inter-tactor distance, stimulation frequency, tactor contact area, burst duration, and stimulus onset asynchrony; however, the limited number of data points precluded definitive conclusions regarding their individual effects. Overall, the strong alignment of data from studies with comparable experimental designs highlights the statistical robustness of our findings.

Finally, we compared our new vibrotactile psychometric functions with reported measures from S-2PD - often cited for its full-body coverage - and static data by Gibson on the forearm with a gap detection method. Static 2PD data are known to be inconsistent and physiologically inapplicable to vibration-based interfaces^25–27^, with thresholds varying by over 200% across studies^24^. Our additional tests for significant differences (Methods 4.5) revealed no discernible patterns among these discrepancies, suggesting that any apparent similarities are likely coincidental, further limiting the relevance of S-2PD for VTD design.

By contrast, non-S-2PD methods, such as the direction-specific data by Gibson and Craig^36^ and the bidirectional data of Tong et al.^25^ closely align despite methodological differences, suggesting greater robustness (SM Fig. 4). A direction specific comparison of Gibson and Craig with our MPFs revealed a positive bias for static acuity in both horizontal and vertical orientations (SM Fig. 1), although significant differences were only observed at select data points. This reinforces the fundamental divergence between static and vibrotactile acuity, emphasizing that a truly data-driven approach to VTD layout must rely on robust vibrotactile measurements - such as those introduced here - rather than static metrics, whose relevance remains limited in dynamic tactile contexts^27^.

### Vibrotactile anisotropy and regional variability

**Fig. 2:**
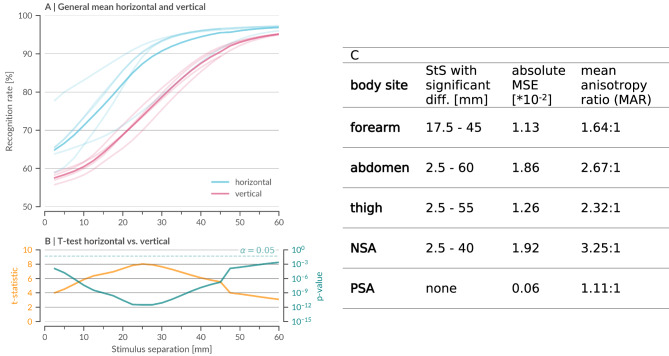
Analysis of general and specific anisotropic vibrotactile spatial acuity. **(A)** General mean psychometric functions from this study (*N* = 33) for horizontal (MPF_h_ blue) and vertical (MPF_v_ red) orientations, computed by averaging all corresponding individual psychometric functions across body sites. Body site-specific MPF_BS_ are shown in lighter shades and color-coded by measurement direction. Standard error is omitted for clarity. **(B)** T-statistic (orange) and logarithmic p-values (teal) from a two-sided t-test with Benjamini–Hochberg correction, performed at each stimulus separation (StS) to assess significant differences between MPF_h_ and MPF_v_. The significance threshold (α = 0.05) is indicated by a dotted line. Horizontal acuity remains significantly higher (*p* < 0.05) across all tested StS. **(C)** Summary of detailed anisotropy measures specific for each of the five body sites (forearm, abdomen, thigh, lower back near-spine area (NSA), and peripheral-spine area (PSA)) in horizontal versus vertical vibrotactile perception (detailed values at Zenodo: statistic_data_fig2c). Stimulus separation ranges (in mm) that showed significant differences for each site determined by a continuous two-sided t-test with Benjamini–Hochberg correction (Methods 4.5). The cumulative mean squared error (MSE) between horizontal and vertical MPF_BS_ quantifies the magnitude of directional divergence, while the mean anisotropy ratio (MAR) captures the relative sensitivity advantage of horizontal perception over a broad range of recognition rates (Methods 4.6). NSA and the abdomen exhibit the strongest anisotropy, whereas PSA shows minimal directional differences. Detailed statistical analyses provided on Zenodo (statistic_data_fig2b).

Building on our robust dataset, we further examined how directional differences shape vibrotactile perception. By comparing MPFs for horizontal and vertical orientations across all tested body sites (Fig. 2A), we observed a consistent horizontal advantage at every stimulus separation. This global trend was statistically validated through independent two-sided t-tests (see Methods 4.5), with corresponding t-statistics and log-scaled p-values corrected for multiple testing (Benjamini-Hochberg) presented in Fig. 2B. Notably, a discontinuity at 45 mm reflects the measurement limit imposed by the forearm.

A more granular, site-specific analysis reinforced these observations. Independent t-tests revealed that all sites, except for the PSA, exhibited significant horizontal–vertical differences over broad ranges of stimulus separations (Fig. 2C). To quantify these disparities, we computed the cumulative mean squared error (MSE) of recognition rate differences at each site. The NSA and abdomen demonstrated the highest mean squared error values, followed by the forearm and thigh, while PSA showed minimal anisotropy.

To capture directional bias more comprehensively, we introduced a mean anisotropy ratio (MAR) that leverages the quasi-continuous MPFs across the full range of stimulus separations (see Methods 4.6). Mean anisotropy ratios highlighted pronounced anisotropy near the body midline - with ratios of 3.25:1 at NSA and 2.67:1 at the abdomen - compared to more moderate ratios at the forearm (1.64:1) and thigh (2.32:1), and a near-unity ratio (1.11:1) at the PSA (see Fig. 2C). Qualitatively consistent with other vibrotactile data^27,31,33^ and static data^36^, these results underscore an intrinsic horizontal advantage in somatosensory processing.

**Fig. 3:**
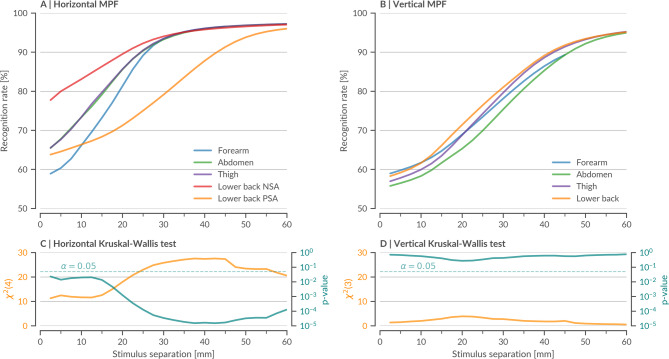
Analysis of regional variability in vibrotactile spatial acuity. Mean psychometric functions (MPFs) from this study (*N* = 33) for body sites, grouped by **(A)** horizontal MPF_BS, h_ and **(B)** vertical measurement MPF_BS, v_ orientations. Color-coding represents the five tested body sites. **(C**,** D)** χ²-statistics (orange) and logarithmic p-values (teal) from a Kruskal–Wallis test with Benjamini–Hochberg correction (Methods 4.5), performed at each stimulus separation (StS) to assess significant differences between MPFs. The significance threshold (α = 0.05) is indicated by a dotted line. Horizontal measurements show significant differences across all StS, whereas vertical measurements exhibit no significant differences. Detailed statistical analyses provided on Zenodo (statistic_data_fig3).

Further analysis compared body site–specific MPFs by orientation using a Kruskal–Wallis test with Benjamini–Hochberg correction at all StS (Fig. 3). We found that horizontal MPFs differed significantly among body sites (χ²(4) > 4; *p* < 0.05; Fig. 3A, C), whereas vertical MPFs clustered more closely with no significant differences (χ²(3) < 3; *p* > 0.05; Fig. 3B, D). Post-hoc analysis identified the NSA and PSA as key contributors, and independent corrected t-tests confirmed that the NSA is significantly more sensitive than the PSA from 2.5 mm to 52.5 mm (SM Fig. 3). Outside the lower back, inter-site differences were modest (SM Fig. 2) - likely reflecting both the lesser sensitive large surface areas studied and vibratory attenuation through subcutaneous tissue. In contrast, the firmer tissue near the spine appears to transmit vibrations more effectively, yielding a pronounced NSA-to-PSA gradient. These findings, consistent with earlier reports of midline regions as perceptual “anchor points”^31,33,37–41^, highlight the importance of incorporating orientation-dependent sensitivity into VTD designs to enhance both efficiency and user performance.

Our stimulation parameters (1 N, ~ 130 Hz) primarily target Pacinian (FAII) corpuscles, which are tuned to vibration in this frequency range. However, quantitative anatomical data on Pacinian density are scarce, especially outside glabrous skin. Corniani & Saal^42^ provide estimates for the overall density of myelinated Aβ tactile afferents (FA and SA combined), reporting values of up to 241 units/cm² in the fingertips and ~ 58 units/cm² in the palm, but only ~ 13 units/cm² on the arms, ~ 10 units/cm² on the legs, and ~ 9 units/cm² on the trunk. Within the hand, Pacinian afferents account for only about 13% of fibers (~ 10–25 units/cm²), and they are described as extremely rare in hairy skin. This overall decline in afferent density outside glabrous regions is consistent with our comparatively low spatial acuity on the arms, abdomen, back, and thigh. Nevertheless, Corniani & Saal^42^ do not provide Pacinian-specific counts for the trunk, and thus cannot account for the pronounced sensitivity gradient we observed between near-spine (NSA) and peripheral (PSA) regions of the lower back. To the best knowledge of the authors, no quantified Pacinian density maps exist for such subregions, which makes a precise neuroscientific contextualization of our results difficult and highlights the need for future anatomical studies of afferent distributions in body areas most relevant for VTD applications.

### Robust vibrotactile display layouts

**Fig. 4:**
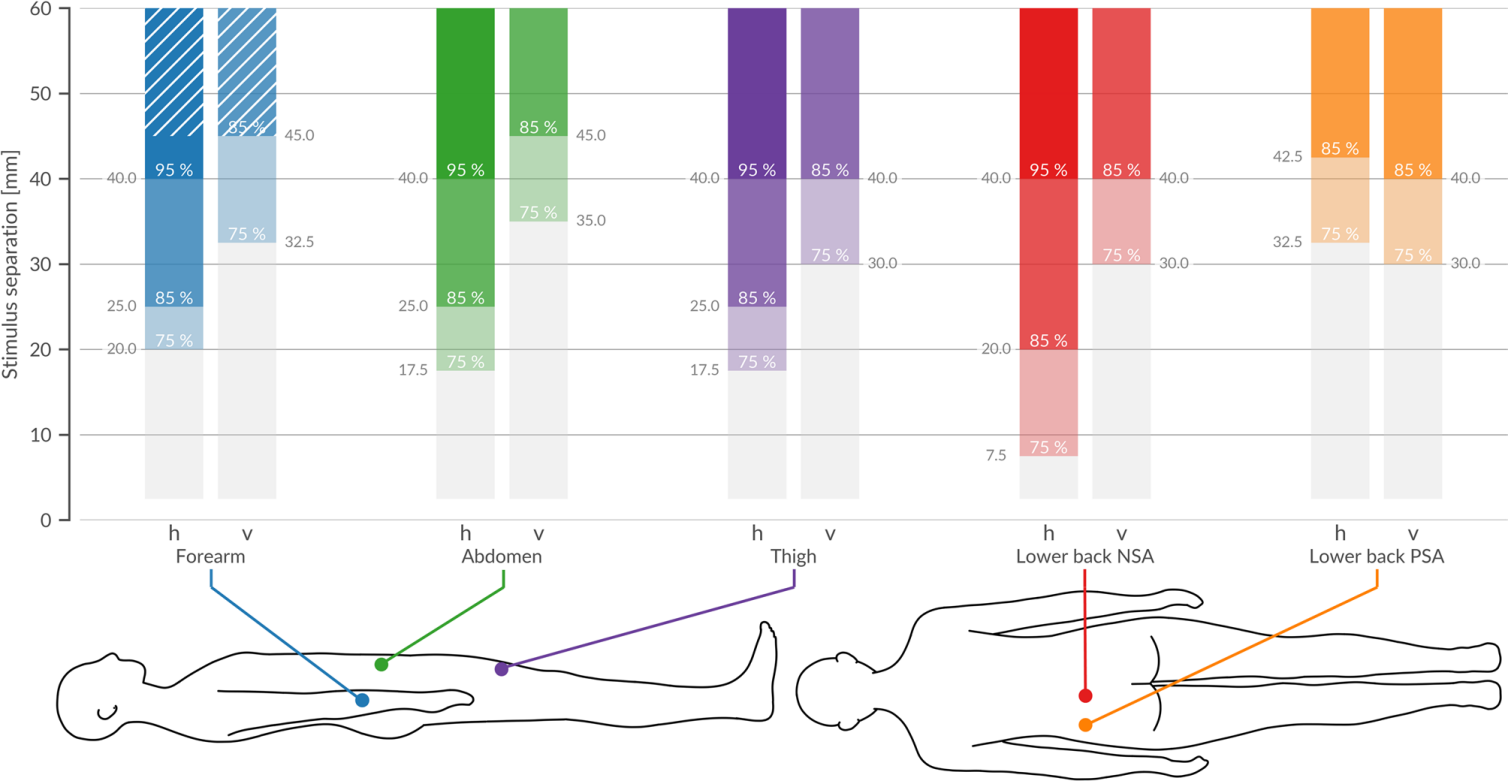
Robust thresholds at three chosen recognition-rate-levels (75, 85, 95%) for each body site and measurement direction. Significant thresholds for each body site (BS) in horizontal and vertical orientations, identified at recognition rates (RR) of 75%, 85%, and 95%. Each bar represents the earliest stimulus separation (StS) at which the selected RR-level is significantly exceeded, as confirmed by a one-sided t-test with Benjamini-Hochberg correction (Methods 4.5). Colors denote the BS, and shading indicates the specific threshold level. In vertical measurements, the 95% threshold is never reached, and the 75% and 85% thresholds appear at substantially larger StS than in the horizontal direction. By contrast, horizontal orientation reveals more pronounced differences, with the lower back near-spine area (NSA) achieving 75% at just 7.5 mm, whereas the peripheral-spine area (PSA) requires 32.5 mm. The forearm’s maximum measured StS is 45 mm, and the 85% threshold is attained vertically at that limit. Since the thresholds are established at the earliest StS meeting significance, no additional variance measures are needed to define them. Detailed statistical analyses provided on Zenodo (statistic_data_fig4).

Figure 4 consolidates our central findings by presenting three statistically validated thresholds - at recognition rates (RR) of 75%, 85%, and 95% - for each body site in both horizontal and vertical orientations. Each colored bar marks the earliest stimulus separation (StS) at which a given RR is confidently reached, as determined by a one-sided one-sample t-test with Benjamini-Hochberg (Methods 4.5) correction. Because the MPFs increase monotonically, these thresholds represent the lowest stimulus separation required to achieve a target RR, offering a statistically robust metric that transcends visual inspection.

The figure also underscores the marked anisotropy in vibrotactile perception. In the vertical orientation, the 75% and 85% threshold occur only at higher StS, with the 95% threshold never reached; moreover, vertical sensitivity remains relatively consistent across body sites. By contrast, horizontal thresholds vary considerably—most notably, NSA requires only 5 mm to reach RR = 75%, while the PSA demands 32.5 mm. On the forearm, the maximum measurable range of 45 mm barely achieves the 85% threshold in the vertical orientation. These observations underscore the need for orientation-aware spacing in VTD design.

From a design perspective, these thresholds provide clear, actionable guidelines for minimal tactor spacing tailored to specific body sites and orientations. The pronounced horizontal acuity, especially near the spine, suggests that more compact tactor layouts can be deployed in these regions, while larger separations are necessary vertically to achieve comparable accuracy. Designers and engineers can derive significant thresholds at any desired RR from our dataset.

**Fig. 5:**
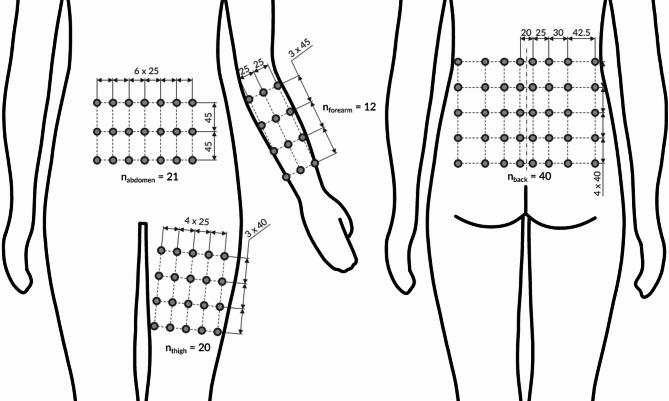
Robust vibrotactile display (VTD) layouts for four large-area body sites. VTD layouts for the forearm, abdomen, thigh, and lower back, created by integrating our significant thresholds with established design insights from the literature^1,43–45^. Each grid denotes potential inter-tactor distances (in mm) and the maximum tactor count (n_BS_​) for each site. At the lower back, the arrangement accommodates the observed horizontal sensitivity gradient by gradually adjusting spacing from near-spine to peripheral regions. While these designs illustrate how our empirical findings and existing guidelines can shape high-density VTD configurations, user-specific calibration may be necessary for optimal performance.

Building on our dataset and current scientific insights, Fig. 5 further illustrates robust VTD layouts for the forearm, abdomen, thigh, and lower back. For this demonstration, we selected inter-tactor distances (ITDs) based on the 85% recognition rate, anticipating that user performance will improve with adaptation over time^46,47^. A cartesian tactor arrangement was employed, allowing spacing adjustments that account for anisotropy - an advantage not afforded by hexagonal layouts^18,19,21^. In our design, vertical spacings were set at 40 mm for the thigh and lower back, and 45 mm for the abdomen and forearm. Horizontally, the forearm, abdomen, and thigh use 25 mm, while the lower back is designed with a variable spacing - from 20 mm near the spine to 42.5 mm in peripheral areas - to reflect the measured sensitivity gradient.

These layouts also incorporate physiological constraints reported in the literature^1,43–45^. Regions such as the abdomen and thigh, which experience stretching, compression, and variable subcutaneous fat distribution, limit the effective area for tactor placement^48^. In contrast, the forearm and lower back, with typically lower fat deposition, yield more reliable vibrotactile acuity^49^. Additionally, areas with minimal soft tissue coverage, such as over the spine, are avoided to prevent discomfort or perceptual artifacts.

Using each site’s homogeneous area and known anatomical restrictions, we further derived maximum tactor counts that indicate the potential complexity of conveyed information, referred to as tactile information transfer^50,51^. Usable areas were estimated via anthropometric data^52,53^, covering the 5th to 95th percentile range. For example, our design suggests 12 tactors for the forearm (arranged in a 3 × 4 grid), 21 tactors for the abdomen (7 × 3), 20 tactors for the thigh (5 × 4), and 40 tactors for the lower back (8 × 5). Beyond these general guidelines, our method supports personalized refinement: a user’s tactile sensitivity profile can be mapped using our apparatus, and these thresholds can then inform a parameterized CAD model for rapid prototyping of individualized VTDs. This direct integration of psychophysical data with device design lays a solid foundation for next-generation tactile interfaces that maximize usability and information throughput.

## Conclusion

We present a robust, data-driven framework for VTD design that overcomes long standing limitations in spatial acuity benchmarks. By generating continuous psychometric functions via Bayesian adaptive parameter estimation—coupled with an apparatus offering infinitely adjustable inter-stimulus distances—we move beyond the previously employed static or single-threshold VT metrics to provide high-resolution, reliable measures across multiple body sites and orientations.

Our extensive dataset, collected from 33 participants, leads to three main conclusions: (1) Previous studies relying on single-value thresholds exhibit significant variability, and static and vibrotactile measures remain fundamentally non-comparable. Our results clarify these disparities and set a new standard for tactile acuity assessment. (2) We reveal a pronounced horizontal advantage over vertical vibrotactile sensitivity, especially near the body midline. This anisotropy necessitates wider vertical spacing in VTD designs to achieve comparable recognition rates. (3) While overall differences across large-area body sites are modest, the lower back demonstrates a clear sensitivity gradient - from 20 mm near the spine to over 40 mm peripherally - underscoring the need for adaptive, site-specific layouts.

These insights directly inform practical VTD layouts for the forearm, abdomen, thigh, and lower back, enabling designers to tailor inter-tactor spacing based on both orientation and anatomical variability. Our approach provides actionable, statistically robust guidelines that promise enhanced performance, comfort, and immersion in tactile interfaces.

Although our study spans five large-area sites, it is limited by its focus on a relatively homogeneous demographic (adults under 38, primarily in an academic setting) and controlled postures. Microanatomical factors - such as skin moisture, hair, subtle morphological variations and other - as well as the use of smaller-contact-area stimulus tips, may further influence outcomes. Another limitation is our deliberate use of fixed stimulus parameters (contact force, vibration frequency, burst duration etc.). This decision ensured statistical robustness and comparability with existing vibrotactile studies and typical VTD applications, but it inevitably reduces the breadth of neuroscientific interpretation. Future work should extend these findings to broader populations and real-world conditions. We plan to apply our derived VTD layouts to wearable assistive devices, refine additional stimulus parameters (e.g., frequency, intensity, temporal modulation), and integrate real-time data encoding techniques (including computer vision) to translate complex spatial data into intuitive tactile signals.

By establishing a validated psychophysical foundation with continuous vibrotactile acuity measures, our study lays the groundwork for high-fidelity, user-centered VTDs. We hope that these advancements will contribute to elevate haptic engineering, ultimately empowering individuals with improved sensory feedback and enhanced quality of life.

## Methods

To address shortcomings in the currently available data (Ch. 2.1), we employed a novel experimental framework^26^ that introduces the following advances in VT spatial acuity measurement: (1) dynamically adjustable tactor spacing, eliminating interpolation; (2) high-resolution stimulus tips for fine-grained measurement at small inter-stimulus distances; (3) Bayesian adaptive parameter estimation to maximize information gain per trial; (4) a fully automated testing process to minimize experimenter bias; and (5) a large participant pool (*N* = 33) with 100 trials per body site, ensuring robust statistical power.

**Fig. 6:**
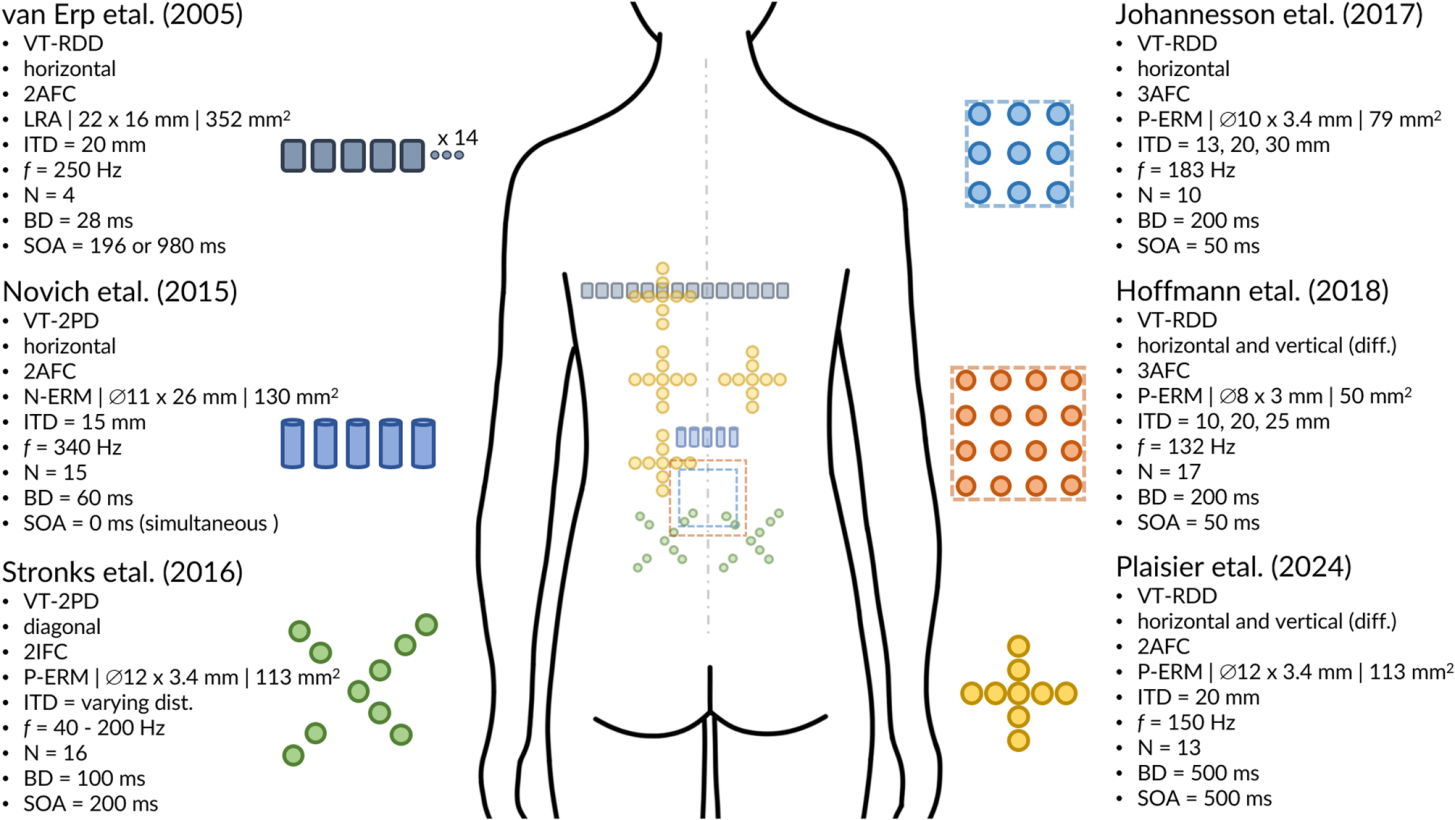
Test parameter and tactor arrangement overview of six prominent studies examining spatial acuity on the back^28,29,31–34^. Each panel illustrates the tactor arrangement body site placement, and key experimental parameters: Task-paradigm, measurement direction, answer-paradigm, tactor (type, measures, contact area), inter tactor distance (ITD), frequency (f), number of participants (N), burst duration (BD), stimulus onset asynchrony (SOA). The placement of each tactor arrangement on the back shown on the silhouette, reflecting the reported measurement sites from the respective studies. Stronks et al.^29^ conducted measurements at two and Plaisier et al.^34^ a four distinct back locations. Further abbreviations: vibrotactile (VT), relative direction discrimination (RDD), two-point discrimination (2PD), linear resonant actuator (LRA), normal eccentric rotating mass vibration motor (N-ERM), parallel eccentric rotating mass vibration motor (P-ERM), two/three alternative forced choice answer paradigm (2AFC/3AFC), two interval forced choice answer paradigm (2IFC). Placement information was extracted to the best of our knowledge from the respective publication.

To contextualize our framework, Fig. 6 summarizes six prominent studies that have examined vibrotactile spatial acuity on the back, highlighting their task paradigms, measurement directions, and key stimulation parameters. These works established essential methodological foundations, yet their diverse designs and limited automation have so far constrained data comparability. Our setup builds upon and refines these approaches by combining their conceptual strengths with enhanced spatial resolution, adaptive control, and full procedural automation.

### Participants

We recruited 33 participants (8 female, aged 18–38, mean age 26.3 ± 4.1 years, mean BMI 22.9 ± 2.0) through university-affiliated social media channels and posters. All participants were compensated financially. None reported medical conditions affecting tactile perception (e.g., diabetes, carpal tunnel syndrome) or cognitive disorders that might influence task performance (e.g., central nervous system disorders, learning disabilities, attention deficits, or dyslexia)^54^. The skin areas tested were free of irritations, dermatitis, tattoos, or scar tissue. All participants provided informed consent and privacy statements prior to testing. This study was approved by the Ethics Committee of the University of Wuppertal and conducted in accordance with the Declaration of Helsinki^55^.

### Sensory testing

**Fig. 7:**
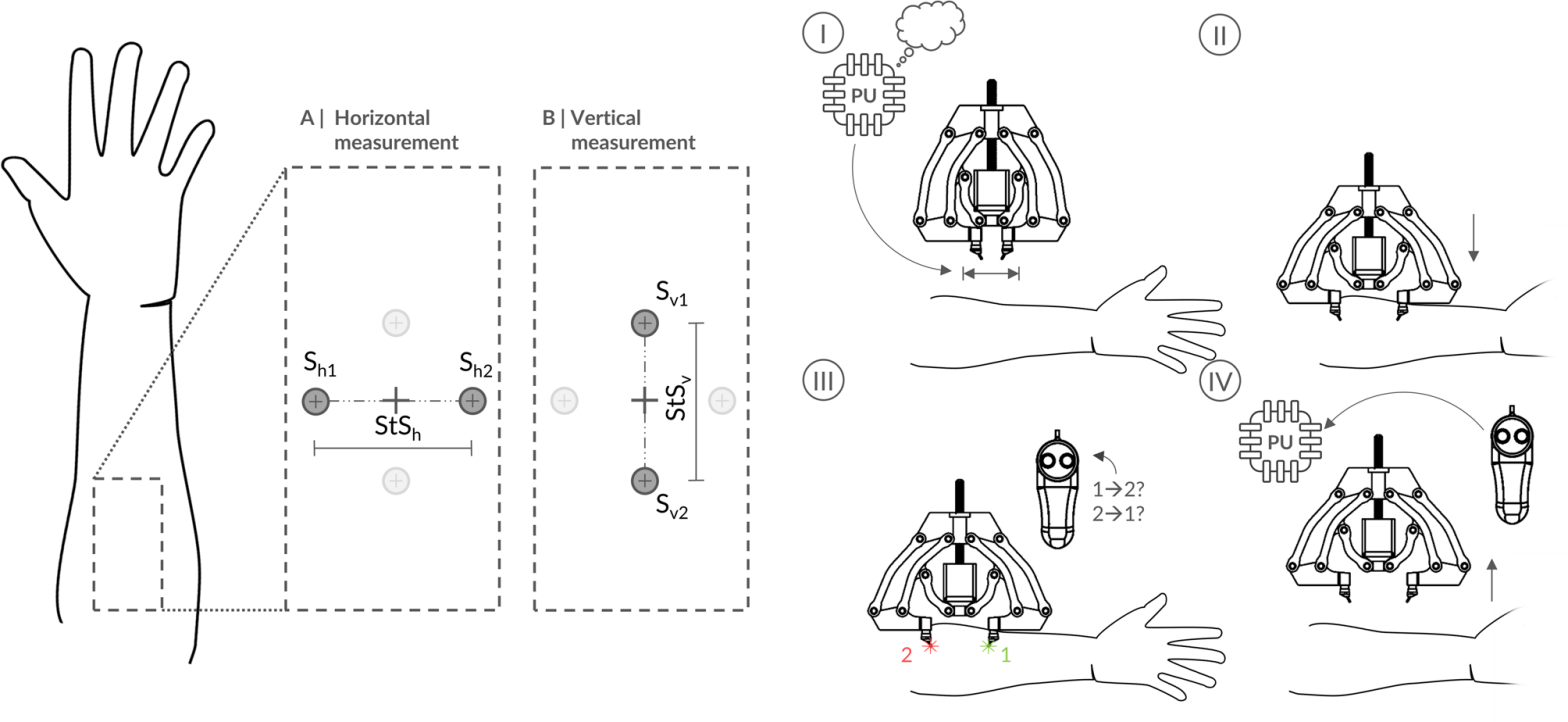
Sensory testing scheme. Illustrative example of the measurement setup on the forearm, showing both horizontal **(A)** and vertical **(B)** orientations of the stimulus tips (S_h1_, S_h2_ and S_v1_, S_v2_), along with their respective stimulus separations (StS_h_​ and StS_v_). In the two-interval forced-choice (2IFC) paradigm, participants identify which tactor vibrated first, indicating whether the stimulus moved from Tip 1 to Tip 2 or vice versa. Four-step testing process loop for each orientation: **(I)** Processing unit (PU) calculates StS to be tested using Bayesian adaptive parameter estimation algorithm (Methods 4.5) and automatically adjusts calipers accordingly. **(II)** Lifter lowers calipers onto the skin. **(III)** Stimuli are presented, and the participant responds via a remote. **(IV)** Calipers are lifted, and the StS, stimulus, and participant’s response are fed back into the algorithm. This loop repeats until termination criterion is met. This setup was replicated for all other body sites in the study. The relative sizes and positions of elements in **I-IV** do not reflect actual proportions.

Task: We employed a two-interval forced-choice (2IFC) paradigm^56^, presenting two successive vibrotactile stimuli and asking participants to indicate which one occurred first. The stimuli were generated by two vibration motors, each driving a stimulus tip (Fig. 7, S_h1_ and S_h2_ for horizontal, S_v1_ and S_v2_ for vertical), separated by the corresponding StS. Throughout testing, both stimulus tips maintained uniform contact with the skin.

Before testing, participants completed a questionnaire covering demographic details, medical history, and prior experience with vibrotactile feedback. They were then seated comfortably for forearm and thigh measurements or positioned on a massage table (prone for lower back and supine for abdomen). To minimize learning effects and fatigue, we randomized the order of body sites and orientations. The apparatus was aligned following a standardized procedure supported by the automated testing program, and participants completed a 10-trial practice session to familiarize themselves with the task and ensure correct alignment. At the start of each trial, the Bayesian adaptive parameter estimation algorithm determined the StS (Fig. 7, I), and the apparatus lowered the stimulus tips onto the skin until a contact force of 1 N (0.5 N per tip) was achieved (II). The stimuli were then presented, and participants responded via the remote (III). After each response, the apparatus retracted, adjusted the calipers to the new StS dictated by the Bayesian algorithm, and repeated the process (IV).

Each orientation (horizontal, vertical) involved 50 trials (~ 9 min), totaling about 18 min per body site. Including setup and short breaks, each body site required 35–40 min of testing. To prevent fatigue, no participant underwent testing on more than two body sites per session.

### Apparatus

**Fig. 8:**
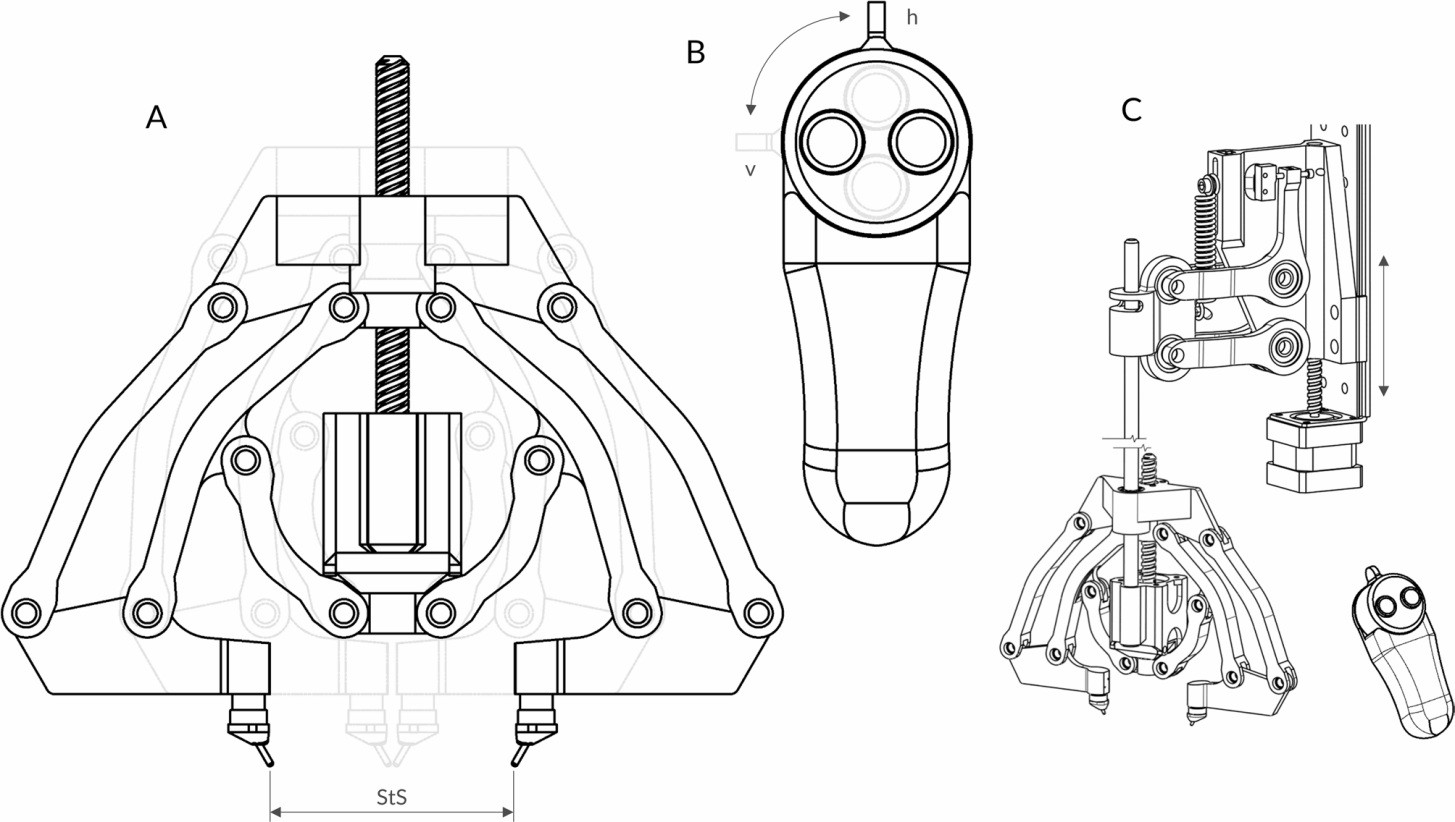
Testing apparatus and remote for participant response. **(A)** Calipers from the testing apparatus shown in two different stimulus separation (StS) configurations (black = 60 mm, blue = 2.5 mm). Two six-bar linkages move the stimulus tips without tilting, driven by an internal stepper motor via a spindle. **(B)** Participant response remote with two orientation configurations for horizontal (h, black) and vertical (v, blue) testing. The rotation is designed to facilitate participant responses and minimize input errors. **(C)** Complete apparatus with calipers mounted on the lifter. The lifter, attached to a rail fixed on a tripod, moves the calipers up and down. Before testing, the experimenter manually adjusts the rail to align the apparatus to the body site. Once positioned, the system operates fully automatically to minimize experimenter bias.

We employed an apparatus originally developed and validated in a previous study^26^, subsequently refined to improve precision and reliability (see Methods 4.3.2). It comprises a lifter mechanism, caliper-based tactor holders, a remote control, and a tripod for stable positioning (Fig. 8). The lifter, equipped with a stepper motor, raises and lowers the calipers that hold two stimulus tips. When the tips contact the skin, a spring mechanism ensures a defined application force of 1 N total (0.5 N per tip, ± 0.03 N), detected by a limit switch. This ensures reproducible contact pressure across trials.

The calipers incorporate two six-point joints to adjust StS with high precision (± 0.1 mm) without rotation or tilt of the stimulus tips. A stepper motor and spindle assembly vary the tactor spacing, allowing continuous adaptation of StS by the Bayesian adaptive parameter estimation algorithm. The participant’s remote control device features two color-coded buttons and a rotatable input panel, which can be aligned to match the orientation of the calipers (horizontal or vertical), thereby minimizing input errors and ensuring intuitive operation.

#### Components and values

As commonly used and validated by Stronks et al.^57^ we selected Precision Microdrives model 310 − 101 (10 × 3.4 mm, 2.5–3.8 V, 1.2 g, 0.8 G) as tactors. Frequency variations between 100 and 250 Hz are generally reported to exhibit minimal impact on sensitivity differences^37,40^, therefore we aimed for a target frequency of ~ 130 Hz^43^. Consistency in frequency profiles for both tactors was prioritized over exactly matching a target frequency. Following Boldt et al.^58^ and Stronks et al.^29^, we set the burst duration (BD) to 200 ms and the stimulus onset asynchrony (SOA) to 500 ms to avoid possible perceptual illusions^59^. The stimulus tips used in conjunction with these tactors had a diameter of 1.5 mm and a contact area of 1.8 mm².

The entire fully automated control and data acquisition system, including the Bayesian adaptive algorithm, was implemented in Python on an NVIDIA Jetson Nano, utilizing Python libraries including numpy^60^, pandas^61^, xarray^62^, matplotlib^63^, seaborn^64^, and psimarginal^65^. CAD models of the experimental apparatus were designed using Onshape^66^ software.

#### Calibration, improvements and further preventive steps for valid results

##### Calibration

**Fig. 9:**
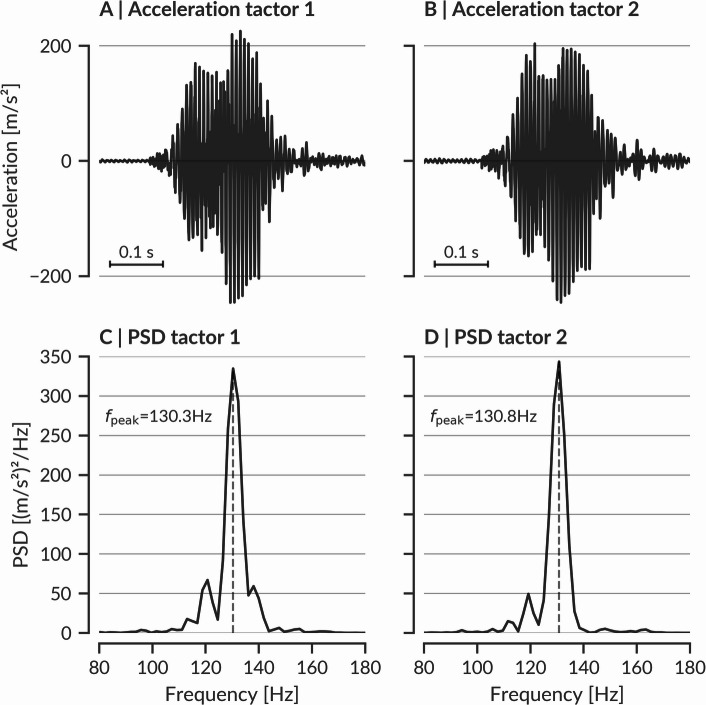
Comparison of vibrational characteristics of tactors after similarity optimization. Vibrational characterization after calibration of the two preselected vibration motors in their mounted state within the apparatus where they function as tactors. **(A)** and **(B)** display the measured acceleration over time for tactor 1 and tactor 2, respectively, recorded at a 2.5 kHz sampling rate. The measurements were conducted at the stimulus tips, which amplify the motor accelerations. Panels **(C)** and **(D)** depict the corresponding power spectral densities (PSDs), revealing closely matched PSD profiles and peak frequencies (≈ 130 Hz) after an iterative optimization process.

Even high-quality eccentric rotating mass (ERM) motors exhibit minor variability in amplitude and frequency due to manufacturing tolerances. Such differences can be amplified by assembly conditions. To prevent participants from relying on subtle intensity differences rather than spatial cues, we first preselected two closely matched motors from a batch of 20. We then calibrated them using a PSV-500 scanning vibrometer^67^, measuring acceleration at the freely oscillating stimulus tips (Fig. 9A, B) at stable environmental conditions (20.5 °C, 45% humidity, 1000 mm measurement distance, 2.5 kHz sampling rate).

We controlled motor output via a 4 V baseline supply voltage. The PWM frequency (30–500 Hz) and duty cycle (DC, 50–95%) were adjusted to match the power spectral densities (PSDs) of both tactors. Using a two-stage optimization (grid-search followed by gradient descent^68^;) and a weighted Euclidean distance metric^69^, we minimized differences in PSD especially within the higher sensitivity 80–350 Hz range. This yielded highly similar PSDs (Fig. 9C and D), and differences were imperceptible to the investigators. Additional measures, including Gaussian noise on motor signals intensity and the use of noise-cancelling headphones with pink noise, further masked any residual differences, ensuring participants could not rely on motor-specific cues. To further prevent passive vibration artifacts, we implemented dampers, reducing passive vibration amplitude from ~ 65% to ~ 35% of the active tactor’s energy density.

### Placement

Figure 4 shows the placement of measurement sites. Each body site was tested in both horizontal (h) and vertical (v) orientations around a common midpoint. Left and right directions are from the participant’s perspective.

- Forearm: We tested the inner, “weak” forearm (left forearm in right-handed individuals vice versa), positioning the midpoint 50–80 mm from the antecubital crease along the forearm’s midline. We selected the widest, flattest region available.
- Abdomen: The midpoint was positioned 20 mm below the navel and 40–50 mm left of the body midline.
- Thigh: The midpoint was placed 180–280 mm above the patella along the seated thigh, ~ two-thirds of the total thigh length from the knee. As with the forearm, a wide, flat area was chosen.
- Lower back NSA: The midpoint aligned with the L2–L3 vertebrae on the erector spinae and lumbar fascia, typically 20–40 mm to the right of the body midline. Few trials required extending the tactor spacing beyond the body midline, as the high sensitivity at small StS values, combined with the Bayesian algorithm’s focus on less certain ranges, rarely prompted large separations at NSA.
- Lower back PSA: For PSA, we tested only horizontal orientation at 50–80 mm right of the midline, again at L2–L3 level. Vertical measurements mirrored NSA conditions due to limited flat surface areas laterally.

### Psychometric evaluation

We determined the StS (stimulus-to-sensation mapping) using a Bayesian adaptive parameter estimation algorithm^25,26,35^, which dynamically selects StS values that maximize information gain. At each trial, stimulus intensities were chosen to minimize expected posterior entropy, refining sampling around critical thresholds. This adaptive approach yields higher data quality and more precise psychometric functions compared to fixed or random StS protocols^35,70^.

We employed Bonferroni-corrected z-tests or t-tests to assess statistical differences between data points from other studies and our own. Normality of distributions was verified using Shapiro–Wilk tests, parametric tests (z-tests, t-tests) were applied only when normality was confirmed. If no significant difference was found, similarity was concluded. Regional variability was analyzed using a Benjamini-Hochberg corrected Kruskal–Wallis test, accounting for non-uniform variances across populations. For comparisons where normality was confirmed, independent one-sided or two-sided t-tests were conducted, with multiple comparisons controlled using the Benjamini–Hochberg procedure (FDR)^71^.

### Formulas

We employed a Bayesian adaptive parameter estimation procedure (as described in Sect. “Psychometric evaluation”) to efficiently estimate each participant’s psychometric function. At every step, we computed posterior probabilities over the parameters of a four-parameter Weibull function with stimulus separations varied in 2.5 mm increments: threshold $\:a$, slope $\:b$, guess rate γ, and lapse rate δ. For each participant, body site, and stimulus orientation, the posterior was derived from a set of 155,000 candidate Weibull curves, systematically covering the entire parameter space. From the resulting posterior distribution, we computed the probability-weighted average curve (postmean**)** which integrates uncertainty about the parameters (details in vom Stein et al.^26^).

$$\:{{\Psi\:}}_{a,b,\gamma\:,\delta\:}\left(x\right)=\gamma\:+(1-\delta\:-\gamma\:)(1-{e}^{{-\left(\frac{x}{a}\right)}^{b}})$$

The mean psychometric function (MPF) for each body site (BS) and direction (horizontal or vertical) was calculated as the average RR of all individual psychometric functions across all participants at each StS:

$$\:{MPF}_{BS,\:h/v}\left(StS\right)=\frac{1}{N}\sum\limits_{i=1}^{N}{RR}_{i}\left(StS\right)$$

where N is the number of participants, and $\:{RR}_{i}\left(StS\right)$ is the recognition rate for participant i at a given StS. This provides a continuous, comparative metric across body sites and directions.

To quantify differences between MPFs across body sites and orientations, we computed the mean squared error (MSE) over the full range of StS in 2.5 mm steps:

$$\:{mse}_{MPFs}=\frac{1}{n}\sum\limits_{i=1}^{n}{\left({MPF}_{1}\left({StS}_{i}\right)-{MPF}_{2}\left({StS}_{i}\right)\right)}^{2}$$

$$\:for\:forearm\:with\:{StS}_{i}\in\:\left\{2.5,\:5,\:7.5,\dots\:,\:45\right\},\qquad\:\:i\in\:\left\{1,\:2,\dots\:,\:18\right\},\:\:\:\:\:n=18$$

$$\:for\:all\:other\:with\:{StS}_{i}\in\:\left\{2.5,\:5,\:7.5,\dots\:,\:60\right\},\qquad\:\:i\in\:\left\{1,\:2,\dots\:,\:24\right\},\:\:\:\:\:n=24$$

Additionally, to assess directional anisotropy, we introduced the mean anisotropy ratio (MAR), building on Gibson and Craig^36^, who compared StS-thresholds as a horizontal-to-vertical ratio at RR = 75% While their method used a single threshold, our MAR extends this across a broader range, leveraging the quasi-continuous nature of our mean MPFs. To derive MAR, we computed ratios for 500 RRs between 0.5 (50% and 1.0 (100%, with a step size of 0.001, for each body site (BS). At each RR_i_​, the interpolated spatial separations StS_h_​ with $\:{{MPF}_{h}}^{-1}\left({RR}_{i}\right)$ and StS_v_ with $\:{{MPF}_{v}}^{-1}\left({RR}_{i}\right)$were extracted, and their ratio was calculated. These ratios we then averaged across the interval, excluding undefined points where ratios could not be computed. This comprehensive method captures directional sensitivity differences across a wider range of thresholds. A higher spatial acuity results in a lower StS at the given RR. If the spatial acuity of MPF_BS, v_ is higher then of MPF_BS, h_, the MAR is above 1 vice versa.

$$\:MAR=\frac{1}{n}\sum\limits_{i=1}^{n}\frac{{{MPF}_{BS,v}}^{-1}\left({RR}_{i}\right)}{{{MPF}_{BS,h}}^{-1}\left({RR}_{i}\right)}$$

$$\:with\:{RR}_{i}\in\:\left\{0.5,\:0.501,\:0.502,\dots\:,\:1\right\},\:\:i\in\:\left\{1,\:2,\dots\:,\:501\right\},\:\:\:\:\:n=501$$

#### Normalization and scaling

To ensure the robustness of our study, we contextualized our findings within the broader body of research on vibrotactile perception. Due to variations in experimental designs and data presentation across studies, direct quantitative comparisons were not readily feasible. Consequently, we normalized the data from other studies to enhance comparability.

Standard error (SE) was derived from reported confidence intervals (CI), standard deviations (SD), or interquartile ranges (IQR) using established statistical transformations^72^:

$\:{CI}_{95}$ given with margin of errors (MoE):

$$\:SE=\frac{MoE}{z}\:\:\:\:\:with\:\:\:\:\:MoE=\frac{{CI}_{0.95}}{2}$$

$$\:for\:{CI}_{0.95}\:with\:z=1.96$$

SD given:

$$\:SE=\frac{SD}{\sqrt{N}}$$

IQR given (Transformation may be imprecise, as IQR is typically used for non-normally distributed data):

$$\:SD=\frac{IQR}{1.349}$$

$$\:SE=\frac{IQR}{1.349\times\:\sqrt{N}}$$

Normalization was applied to studies with a TGR differing from ours (TGR = 50%^73^, details in SM Table 1) by scaling the mean RR relative to the respective TGR, as determined by the specific forced-choice paradigm used. We assumed that all studies followed normally distributed random variables.

$$\:{{RR}_{n}=TGR}_{n}+({RR}_{g}-{TGR}_{g})\frac{(1-{TGR}_{n})}{(1-{TGR}_{g})}$$

$$\:{SE}_{n}={SE}_{g}\frac{(1-{TGR}_{n})}{(1-{TGR}_{g})}$$

## Supplementary Information

Below is the link to the electronic supplementary material.

Supplementary Material 1

## Data Availability

The datasets we generated and analyzed during the current study, including detailed results of statistical analyses, are openly available at Zenodo: https://doi.org/10.5281/zenodo.17295435.The dataset also includes participant metadata (biological sex, age, height, weight), which we collected but did not analyze in the present study.

## References

[1] Chai, C. et al. When to use vibrotactile displays? A meta-analysis for the role of vibrotactile displays in human-computer interaction. *Appl. Ergon.* **103**, 103802 (2022). 35623202 10.1016/j.apergo.2022.103802

[2] Eagleman, D. M. & Perrotta, M. V. The future of sensory substitution, addition, and expansion via haptic devices. *Front. Hum. Neurosci.* **16**10.3389/fnhum.2022.1055546 (2023). 10.3389/fnhum.2022.1055546 PMC9880183 36712151

[3] Friedrich, D. T. et al. Features of haptic and tactile feedback in TORS-a comparison of available surgical systems. *J. Robotic Surg.* **12**, 103–108. 10.1007/s11701-017-0702-4 (2018). 10.1007/s11701-017-0702-4 28470408

[4] Colan, J., Davila, A. & Hasegawa, Y. A review on tactile displays for conventional laparoscopic surgery. *Surgeries* **3**, 334–346. 10.3390/surgeries3040036 (2022).

[5] Thomas, N., Fazlollahi, F., Brown, J. D. & Kuchenbecker, K. J. Sensorimotor-inspired Tactile Feedback and Control Improve Consistency of Prosthesis Manipulation in the Absence of Direct Vision. In *2021 IEEE/RSJ International Conference on Intelligent Robots and Systems (IROS)* (IEEE pp. 6174–6181. (2021).

[6] Vargas, L., Huang, H., Zhu, Y., Kamper, D. & Hu, X. Resembled tactile feedback for object recognition using a prosthetic hand. *IEEE Robot Autom. Lett.* **7**, 10977–10984 (2022). 10.1109/lra.2021.3122897 PMC9248871 35784093

[7] van Erp, J. B. F., van Veen, H. A. H. C., Jansen, C. & Dobbins, T. Waypoint navigation with a vibrotactile waist belt. *ACM Trans. Appl. Percept.* **2**, 106–117. 10.1145/1060581.1060585 (2005).

[8] Perrotta, M. V., Asgeirsdottir, T. & Eagleman, D. M. Deciphering sounds through patterns of vibration on the skin. *Neuroscience* **458**, 77–86. 10.1016/j.neuroscience.2021.01.008 (2021). 33465416 10.1016/j.neuroscience.2021.01.008

[9] Li Pira, G., Aquilini, B., Davoli, A., Grandi, S. & Ruini, C. The use of virtual reality interventions to promote positive mental health: systematic literature review. *JMIR Mental Health*. **10**, e44998. 10.2196/44998 (2023). 37410520 10.2196/44998 PMC10360019

[10] Lie, S. S., Helle, N., Sletteland, N. V., Vikman, M. D. & Bonsaksen, T. Implementation of virtual reality in health professions education: scoping review. *JMIR Med. Educ.* **9**, e41589. 10.2196/41589 (2023). 36692934 10.2196/41589 PMC9906320

[11] Ntakakis, G. et al. Exploring the use of virtual reality in surgical education. *World J. Transplantation*. **13**, 36–43. 10.5500/wjt.v13.i2.36 (2023). 10.5500/wjt.v13.i2.36 PMC9993190 36908307

[12] Sun, Z., Zhu, M., Shan, X. & Lee, C. Augmented tactile-perception and haptic-feedback rings as human-machine interfaces aiming for immersive interactions. *Nat. Commun.* **13**, 5224. 10.1038/s41467-022-32745-8 (2022). 36064838 10.1038/s41467-022-32745-8 PMC9445040

[13] Stanke, D., Duente, T., Rohs, M. & TactileWear: A Comparison of Electrotactile and Vibrotactile Feedback on the Wrist and Ring Finger. In *Proceedings of the 11th Nordic Conference on Human-Computer Interaction: Shaping Experiences, Shaping Society*, edited by D. Lamas, H. Sarapuu, I. Šmorgun & G. BergetACM, New York, NY, USA, pp. 1–13. (2020).

[14] Yadav, N., Sadeghi, N. & Kang, J. Five factors affecting the on-body placement of wearable tactile safety promotion device for construction workers-on-foot. *CI* **24**, 537–557 (2024).

[15] Zhang, Z., Lo, W. H. & Huang, G. The Impact of Meaningful Vibrotactile Displays on User Preferences Across Age Groups in Automated Driving. *Proceedings of the Human Factors and Ergonomics Society Annual Meeting*; (2024). 10.1177/10711813241275919

[16] Yu, X. et al. Skin-integrated wireless haptic interfaces for virtual and augmented reality. *Nature* **575**, 473–479 (2019). 31748722 10.1038/s41586-019-1687-0

[17] Jung, Y. H., Kim, J. H. & Rogers, J. A. Skin-Integrated Vibrohaptic Interfaces for Virtual and Augmented Reality. *Adv. Funct. Mater.* **31**, 10.1002/adfm.202008805 (2021).

[18] Jung, Y. H. et al. A wireless haptic interface for programmable patterns of touch across large areas of the skin. *Nat. Electron.* **5**, 374–385. 10.1038/s41928-022-00765-3 (2022).

[19] Martinez, J. S., Tan, H. Z. & Cholewiak, R. W. Psychophysical Studies of Interleaving Narrowband Tactile Stimuli to Achieve Broadband Perceptual Effects. *Front. Virtual Real.* **3**, 10.3389/frvir.2022.894575 (2022).

[20] Huang, Y. et al. A skin-integrated multimodal haptic interface for immersive tactile feedback. *Nat. Electron.* **6**(12), 1020-1031 (2023).

[21] Flavin, M. T. et al. Bioelastic state recovery for haptic sensory substitution. *Nature* **635**, 345–352 (2024). 39506124 10.1038/s41586-024-08155-9

[22] Weber, E. H., Helen, E. R. & David, J. M. *E.H. Weber on the Tactile Senses (translated from Original 1834)* (Psychology Press, 1834/2018).

[23] Weinstein, S. Intensive and extensive aspects of tactile sensitivity as a function of body part, sex, and laterality. *The Skin Senses: Proceedings*, 195–222 (1968).

[24] Mancini, F. et al. Whole-body mapping of Spatial acuity for pain and touch. *Ann. Neurol.* **75**, 917–924 (2014). 24816757 10.1002/ana.24179 PMC4143958

[25] Tong, J., Mao, O. & Goldreich, D. Two-point orientation discrimination versus the traditional two-point test for tactile Spatial acuity assessment. *Front. Hum. Neurosci.* **7**, 579. 10.3389/fnhum.2013.00579 (2013). 24062677 10.3389/fnhum.2013.00579 PMC3772339

[26] vom Stein, M., Hoppe, M., Sommer, M. & Wolf, K. D. Measuring the Spatial acuity of vibrotactile stimuli: A new approach to determine universal and individual thresholds. *Displays* **80**, 102546. 10.1016/j.displa.2023.102546 (2023).

[27] Jouybari, A. F., Franza, M., Kannape, O. A., Hara, M. & Blanke, O. Tactile Spatial discrimination on the torso using vibrotactile and force stimulation. *Exp. Brain Res.* **239**, 3175–3188. 10.1007/s00221-021-06181-x (2021). 34424361 10.1007/s00221-021-06181-x PMC8541989

[28] Novich, S. D. & Eagleman, D. M. Using space and time to encode vibrotactile information: toward an estimate of the skin’s achievable throughput. *Exp. Brain Res.* **233**, 2777–2788. 10.1007/s00221-015-4346-1 (2015). 26080756 10.1007/s00221-015-4346-1

[29] Stronks, H. C., Parker, D. J. & Barnes, N. Vibrotactile Spatial acuity and intensity discrimination on the lower back using coin motors. *IEEE Trans. Haptics*. **9**, 446–454 (2016). 27214917 10.1109/TOH.2016.2569484

[30] Elsayed, H. et al. VibroMap. *Proc. ACM Interact. Mob. Wearable Ubiquitous Technol.* **4**, 1–16 (2020). 34651096 10.1145/3380987 PMC8513752

[31] van Erp, J. Vibrotactile Spatial Acuity on the Torso: Effects of Location and Timing Parameters. In *First Joint Eurohaptics Conference and Symposium on Haptic Interfaces for Virtual Environment and Teleoperator Systems* (IEEE2005) pp. 80–85. .

[32] Jóhannesson, Ó. I. et al. Relative vibrotactile Spatial acuity of the torso. *Exp. Brain Res.* **235**, 3505–3515. 10.1007/s00221-017-5073-6 (2017). 28856387 10.1007/s00221-017-5073-6 PMC5649388

[33] Hoffmann, R., Valgeirsdóttir, V. V., Jóhannesson, Ó. I., Unnthorsson, R. & Kristjánsson, Á. Measuring relative vibrotactile Spatial acuity: effects of tactor type, anchor points and tactile anisotropy. *Exp. Brain Res.* **236**, 3405–3416. 10.1007/s00221-018-5387-z (2018). 30293171 10.1007/s00221-018-5387-z PMC6267683

[34] Plaisier, M. A., Vos, W. K., Kappers, A. M. L. & others. Vibrotactile spatial acuity on the back. *Perception*, **53**(9), 10.1177/03010066241258969, 619-631 (2024). 38863276 10.1177/03010066241258969 PMC11348621

[35] Kontsevich, L. L. & Tyler, C. W. Bayesian adaptive Estimation of psychometric slope and threshold. *Vision. Res.* **39**, 2729–2737. 10.1016/s0042-6989(98)00285-5 (1999). 10492833 10.1016/s0042-6989(98)00285-5

[36] Gibson, G. O. & Craig, J. C. Tactile Spatial sensitivity and anisotropy. *Percept. Psychophys.* **67**, 1061–1079. 10.3758/bf03193632 (2005). 16396014 10.3758/bf03193632

[37] Cholewiak, R. W. & Collins, A. A. Vibrotactile localization on the arm: effects of place, space, and age. *Percept. Psychophys.* 1058–1077. 10.3758/BF03194834 (2003). 10.3758/bf03194834 14674633

[38] Plaisier, M. A., Sap, L. I. N. & Kappers, A. M. L. Perception of vibrotactile distance on the back. *Sci. Rep.* **10**, 17876. 10.1038/s41598-020-74835-x (2020). 33087741 10.1038/s41598-020-74835-x PMC7577989

[39] Yeganeh, N., Makarov, I., Stefánsson Thors, S. S., Kristjánsson, Á. & Unnthorsson, R. Evaluating the Optimum Distance between Voice Coil Actuators Using the Relative Point Localization Method on the Forearm. *Actuators* **12**, 6. 10.3390/act12010006 (2023).

[40] Yeganeh, N., Makarov, I., Unnthorsson, R. & Kristjánsson, Á. Effects of stimulus frequency and location on vibrotactile discrimination performance using voice coil actuators on the forearm. *Actuators* **12**, 224. 10.3390/act12060224 (2023).

[41] Pratt, S. et al. Tactile localization accuracy at the low back. *Atten. Percept. Psychophysics*. **86**, 1008–1021 (2024). 10.3758/s13414-024-02843-4 PMC11062953 38332382

[42] Corniani, G. & Saal, H. P. Tactile innervation densities across the whole body. *J. Neurophysiol.* **124**, 1229–1240. 10.1152/jn.00313.2020 (2020). 32965159 10.1152/jn.00313.2020

[43] Gescheider, G. A. *Tactile Psychophysics* (Psychology Press, 2008).

[44] Jones, L. A. & Smith, A. M. Tactile sensory system: encoding from the periphery to the cortex. *Wiley Interdiscip. Rev. Syst. Biol. Med.* **6**, 279–287. 10.1002/wsbm.1267 (2014). 24648403 10.1002/wsbm.1267

[45] Shao, Y. *Tactile Sensing, Information, and Feedback Via Wave Propagation* (Springer International Publishing AG, 2022).

[46] Jones, L. A., Kunkel, J. & Piateski, E. Vibrotactile pattern recognition on the arm and back. *Perception* **38**, 52–68 (2009). 19323136 10.1068/p5914

[47] Stronks, H. C., Walker, J., Parker, D. J. & Barnes, N. Training improves vibrotactile Spatial acuity and intensity discrimination on the lower back using coin motors. *Artif. Organs*. **41**, 1059–1070. 10.1111/aor.12882 (2017). 28569046 10.1111/aor.12882

[48] Shah, V. A., Casadio, M., Scheidt, R. A. & Mrotek, L. A. Vibration propagation on the skin of the arm. *Appl. Sci. (Basel Switzerland)*. **9**10.3390/app9204329 (2019). 10.3390/app9204329 PMC8493869 34621542

[49] Björntorp, P. The regulation of adipose tissue distribution in humans. *Int. J. Obes. Relat. Metabolic Disorders: J. Int. Association Study Obes.* **20**, 291–302 (1996). 8680455

[50] Tan, H. Z., Reed, C. M. & Durlach, N. I. Optimum information transfer rates for communication through haptic and other sensory modalities. *IEEE Trans. Haptics*. **3**, 98–108 (2010). 27788117 10.1109/TOH.2009.46

[51] Tan, H. Z., Choi, S., Lau, F. W. Y. & Abnousi, F. Methodology for maximizing information transmission of haptic devices: A survey. *Proc. IEEE*. **108**, 945–965 (2020).

[52] O’Sullivan, S. B. et al. (eds) *Physical rehabilitation* (F.A. Davis, 2019).

[53] Bonin, D., Ackermann, A., Radke, D., Peters, M. & Wischniewski, S. Anthropometric dataset for the German working-age population using 3D body scans from a regional epidemiological health study and a weighting algorithm. *Ergonomics* **66**, 1057–1071. 10.1080/00140139.2022.2130440 (2023). 36226532 10.1080/00140139.2022.2130440

[54] Grant, A. C., Zangaladze, A., Thiagarajah, M. C. & Sathian, K. Tactile perception in developmental dyslexia: a psychophysical study using gratings. *Neuropsychologia* **37**, 1201–1211. 10.1016/s0028-3932( (1999). 99)00013 – 5. 10509841 10.1016/s0028-3932(99)00013-5

[55] Bibbins-Domingo, K., Brubaker, L. & Curfman, G. The 2024 revision to the declaration of helsinki: modern ethics for medical research. *JAMA* **333**, 30–31. 10.1001/jama.2024.22530 (2025). 39425945 10.1001/jama.2024.22530

[56] Duarte, F., Figueroa, T. & Lemus, L. A Two-interval Forced-choice task for multisensory comparisons. *J. Visualized Experiments: JoVE*. 10.3791/58408 (2018). 30474635 10.3791/58408

[57] Stronks, H. C., Parker, D. J., Walker, J., Lieby, P. & Barnes, N. The feasibility of coin motors for use in a vibrotactile display for the blind. *Artif. Organs*. **39**, 480–491. 10.1111/aor.12414 (2015). 25586668 10.1111/aor.12414

[58] Boldt, R., Gogulski, J., Gúzman-Lopéz, J., Carlson, S. & Pertovaara, A. Two-point tactile discrimination ability is influenced by Temporal features of stimulation. *Exp. Brain Res.* **232**, 2179–2185 (2014). 24668131 10.1007/s00221-014-3908-y

[59] Goldreich, D. & Tong, J. Prediction, postdiction, and perceptual length contraction: a bayesian low-speed prior captures the cutaneous rabbit and related illusions. *Front. Psychol.* **4**, 221. 10.3389/fpsyg.2013.00221 (2013). 23675360 10.3389/fpsyg.2013.00221 PMC3650428

[60] Harris, C. R. et al. Array programming with numpy. *Nature* **585**, 357–362. 10.1038/s41586-020-2649-2 (2020). 32939066 10.1038/s41586-020-2649-2 PMC7759461

[61] The pandas development team. pandas-dev/pandas: Pandas. Zenodo, (2024).

[62] Hoyer, S., Hamman, J. & xarray,. xarray: N-D labeled Arrays and Datasets in Python. *JORS*. **5**, 10. 10.5334/jors.148 (2017).

[63] Hunter, J. D. & Matplotlib A 2D graphics environment. *Comput. Sci. Eng.* **9**, 90–95. 10.1109/MCSE.2007.55 (2007).

[64] Waskom, M. Seaborn: statistical data visualization. *JOSS* **6**, 3021. 10.21105/joss.03021 (2021).

[65] Nynke Niehof, J. C. *PsiMarginal. Psi adaptive staircase procedure for use in psychophysic*,.

[66] Onshape *Onshape,*

[67] Polytec GmbH. PSV-500 Scanning Vibrometer. (2024). Available at https://www.polytec.com/de/vibrometrie/produkte/full-field-vibrometer/psv-500-scanning-vibrometer

[68] Nocedal, J. & Wright, S. J. *Numerical optimization* (Springer, 2006).

[69] Duda, R. O., Hart, P. E. & Stork, D. G. *Pattern classification* (John Wiley & Sons Inc, 2001).

[70] Prins, N. Easy, bias-free bayesian hierarchical modeling of the psychometric function using the Palamedes toolbox. *Behav. Res. Methods*. **56**, 485–499. 10.3758/s13428-023-02061-0 (2024). 36703004 10.3758/s13428-023-02061-0

[71] Montgomery, D. C. *Design and analysis of experiments* (Wiley, 2017).

[72] Altman, D. G. *Practical statistics for medical research* (Chapman & Hall/CRC, 1999).

[73] J. Kevin O’Regan, Alva No&euml. A sensorimotor account of vision and visual consciousness. 10.1017/s0140525x01000115 12239892

