# Supplementary material for: Data-driven design guide for vibrotactile display layouts by continuous mapping
Source: Sci Rep. 2025 Nov 12;15:39579. doi: 10.1038/s41598-025-25416-3 (PMC12612159; doi:10.1038/s41598-025-25416-3)
Supplement: Supplementary file 1 — Supplementary Material 1 [file 41598_2025_25416_MOESM1_ESM.pdf]

Supplementary Material

for Data-driven design guide for vibrotactile display layouts by continuous mapping
by vom Stein, M., Hoppe, M., Rieger, N., Wolf, K.-D.

Datasets, raw and processed data, as well as detailed statistical results, are openly available at Zenodo: https://doi.org/10.5281/zenodo.17295435

Supplementary Material Tab. 1 | Overview of scaling and normalization procedures applied to different studies for approximate alignment with our data.
Abbreviations: vibrotactile (VT), relative direction discrimination (RDD), two-point discrimination (2PD), stimulus separation (StS), recognition rate (RR), theoretical guess rate (TGR), standard error (SE), confidence interval (CI), interquartile range (IQR), two/three alternative forced choice (2AFC/3AFC), two interval forced choice (2IFC). The table indicates how we converted each study's reported measures (e.g., 95 % CI, SD, IQR) to SE and subsequently scaled according to the appropriate TGR (typically 50 % for 2AFC or 33.3 % for 3AFC answer paradigms). This approach enables the comparison of studies with varying paradigm. Elsayed et al.[1] employed two methods: a hyperlocalization task, which is not directly comparable as VTD design relies on relative localization, and a VT-2PD assessment, which showed the largest deviations among referenced studies, placing it beyond the plotted range. Stronks et al.[2] employed a variation of the VT-2PD paradigm using a 2IFC design. In each trial, they randomly presented two stimulus variations - one with both tactors activated and one with only a single tactor activated - and participants were required to identify the dual-tactor stimulus. This approach resulted in a theoretical guess rate (TGR) of 50 %, contrasting with the typical VT-2PD protocol, which assumes a TGR of 0 %.

**Reference**	**given**			**calculated**	
	**Result Type**	**Dispersion Metrics**	**TGR**	**Stat. transformation**	**TGR normalization**
**van Erp et al.**<br>VT-RDD	Mean StS<br>@ 75 % RR	95 % CI	50 %	95 % CI → SE	none
**Novich et al.**<br>VT-2PD	Mean RR<br>@ StS-Level	SE	0 %	none	RR + SE<br>0 % → 50 % TGR
**Stronks et al.**<br>VT-2PD (2IFC)	Mean StS<br>@ 75 % RR	SD	50 %	SD → SE	none
**Johannesson et al.**<br>VT-RDD	Mean RR<br>@ StS-Level	SD	33.3 %	SD -> SE	RR + SE<br>33.3% → 50 % TGR
**Hoffmann et al.**<br>VT-RDD	Mean RR<br>@ StS-Level	SE	33.3 %	none	RR + SE<br>33.3 % → 50 % TGR
**Elsayed et al.**<br>VT-2PD	Mean StS<br>@ 50 % RR	95 % CI	0 %	out of bounds	out of bounds
**Plaisier et al.**<br>VT-RDD	Median StS<br>@ 84 % RR	IQR	50 %	IQR -> SE	none

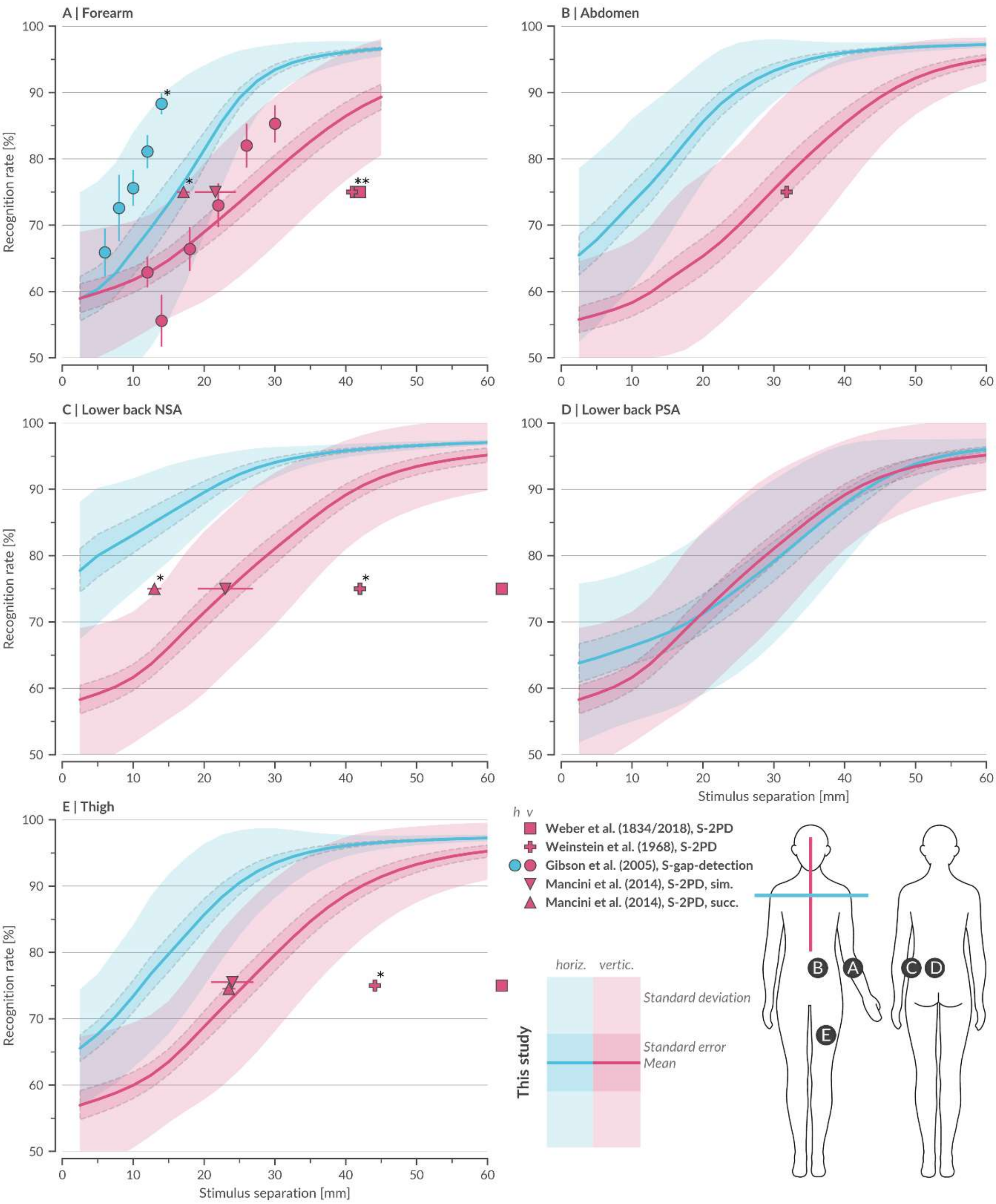

Supplementary Material Fig. 1 | Mean psychometric functions of vibrotactile spatial acuity across body sites alongside static data from reference literature.
Mean psychometric functions (MPFs) from this study (N = 33) showing recognition rate as a function of stimulus separation for five body sites (BS, A–E), measured in both horizontal (blue) and vertical (red) orientations, with ±1 standard error and ±1 standard deviation. Forearm measurements were limited to 45 mm due to space constraints. Static data points from four reference studies[3–6] (data at Zenodo: ref_data_static) presented with ±1 standard error as x- or y-errors, color-coded for horizontal and vertical thresholds. Reference data points were tested for significant difference from our data using Bonferroni-corrected z-tests or t-tests (marked with *, Methods 4.5). Mancini et al.[6] applied the static two point discrimination (S-2PD) method in two variants using either simultaneous (▼) or successive stimuli (▲). Detailed statistical analyses provided on Zenodo (statistic_data_SM_fig1).

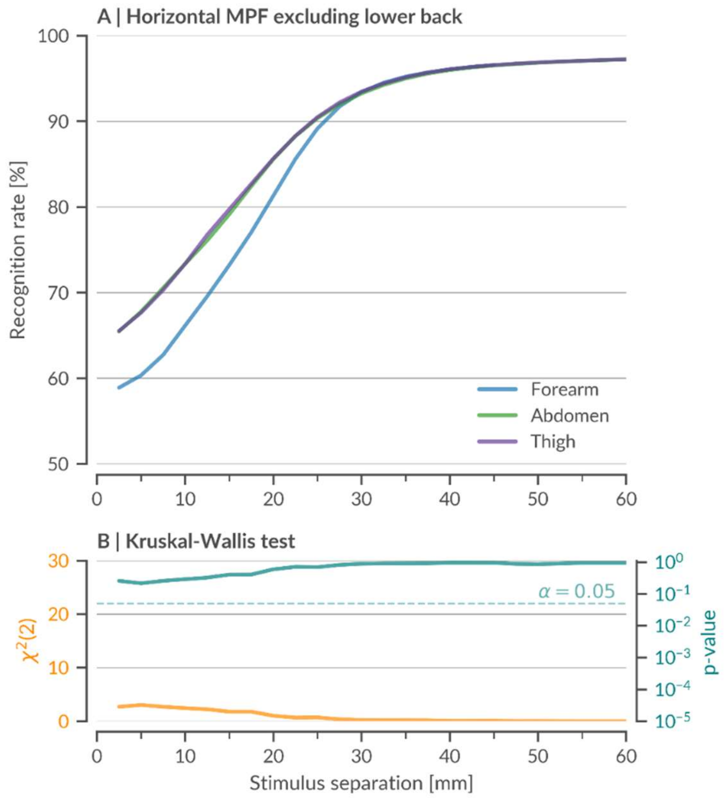

Supplementary Material Fig. 2 | Post-hoc analysis of regional variability of vibrotactile acuity in the horizontal orientation after excluding both lower back sites.

(A) Mean psychometric functions $MPF_{BS,h}$ for the forearm, abdomen, and thigh, revealing minimal differences in recognition rates among remaining body sites. (B) Continuous Kruskal–Wallis test with Benjamini-Hochberg correction (orange, Methods 4.5) and logarithmically scaled p-value (teal). The dashed line indicates α=0.05. In the absence of the lower back data, no significant discrepancies persist, confirming that the previously observed differences were driven by the near-spine and peripheral-spine regions. Detailed statistical analyses provided on Zenodo (statistic_data_SM_fig2).

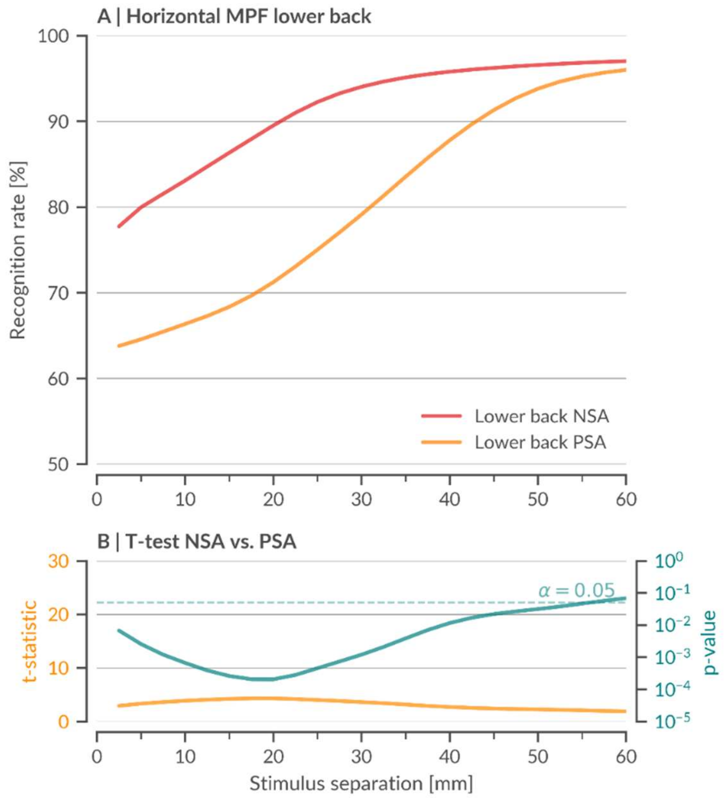

Supplementary Material Fig. 3 | Post-hoc analysis of regional variability for lower back in horizontal orientation revealing sensitivity gradient.

(A) Mean psychometric functions $MPF_{BS,h}$ for near-spine area (NSA) and peripheral-spine area (PSA), highlighting pronounced differences in recognition rates. (B) Continuous independent Benjamini–Hochberg corrected two-sided t-tests (Methods 4.5), with t-statistics (orange) and logarithmically scaled p-values (teal). The dashed line indicates $\alpha = 0.05$. NSA and PSA differ significantly for stimulus separations (StS) between 2.5 mm and 52.5 mm, where the corrected p-value surpasses 0.05. Detailed statistical analyses provided on Zenodo (statistic_data_SM_fig3).

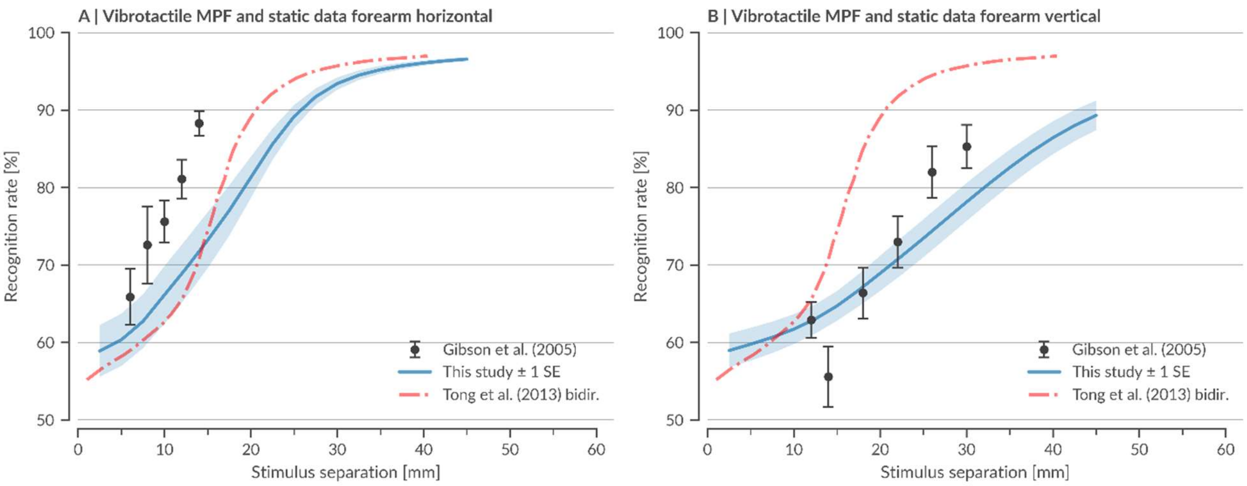

Supplementary Material Fig. 4 | Mean psychometric functions of vibrotactile spatial acuity at forearm alongside static data from reference literature.

MPF from this study in horizontal (A) and vertical (B) measurement direction. Alongside static spatial acuity data from Tong et al.[7] (red dotted line, no SE) and Gibson et al.[5] (black spheres ± 1 SE) are shown for comparison. Data by Tong et al. were measured bidirectionally and therefore do not distinguish between horizontal and vertical orientations.

Glossary

Vocab	*Explanation*
Sensory Substitution, Sensory Expansion, Sensory Addition	Concepts referring to the replacement, enhancement, or supplementation of sensory modalities, often explored in tactile feedback systems[8]
Measurement Directions	Defined in a neutral standing posture[9]:<br>**Horizontal**: Medial–lateral axis, orthogonal to the body midline<br>**Vertical**: For extremities, the proximal–distal axis; for the torso, the cranial–caudal axis, parallel to the body midline
BS (Body Site)	The anatomical areas where measurements were conducted
Stimulus Types	**Static stimuli**: Non-oscillatory tactile inputs delivered through sustained contact with the skin, often referred to as pressure or force stimuli<br>**Vibrotactile stimuli**: Inputs characterized by oscillatory (vibratory) motion of the contact surface<br>**Electric stimuli**: Inputs delivered through electrical impulses applied to the skin
XAFC (X Alternative Forced Choice)	A paradigm where participants must select one option from X alternatives, with no option for abstention or uncertainty. Commonly used are 2AFC and 3AFC paradigms. In a 2AFC (Two-Alternative Forced Choice) task, participants choose between two spatially or contextually distinct stimuli. For example, a vibrotactile stimulus may be presented on either the left or right hand, requiring the participant to identify its location. Unlike paradigms with temporal separation, both options exist simultaneously or differ in content.
XIFC (X Interval Forced Choice)	Similar to AFC, except that all stimuli options are presented sequentially, and the participant reports their temporal order. In a 2IFC (Two-Interval Forced Choice) task, two stimuli are presented in separate time intervals, and participants must determine in which interval a specific feature occurred (e.g., which stimulus was stronger or appeared first). Unlike AFC, where choices exist simultaneously, 2IFC introduces a temporal separation between alternatives.
P-ERM (Parallel Eccentric Rotating Mass)	A type of vibration motor where an eccentric mass rotates parallel to the contact surface, commonly seen in coin motors
N-ERM (Normal Eccentric Rotating Mass)	A vibration motor where an eccentric mass rotates orthogonal to the contact surface, commonly seen in cylindrical motors
LRA (Linear Resonant Actuator)	A vibration motor that drives an inherent mass using an oscillating magnetic field

StS (Stimulus Separation)	The distance between two stimuli presented for discrimination
RR (Recognition Rate)	The proportion of correct responses to a given tactile stimulus (between 0 and 1)
TGR (Theoretical Guess Rate)	The recognition rate expected under unbiased random guessing. Based on the response paradigm (e.g., 2AFC/2IFC TGR = 0.5; 3AFC/3IFC TGR = $0.\overline{3}$)
EGR (Empirical Guess Rate)	The recognition rate observed at the smallest stimulus separation (StS) in empirical data. If this significantly exceeds the TGR, it indicates a methodological bias (e.g., in traditional 2PD paradigms)
PF (Psychometric Function)	A sigmoidal function used to model perceptual performance, mapping recognition rates across varying stimulus separations
IPF (Individual Psychometric Function)	A psychometric function fitted to data from a single participant
MPF (Mean Psychometric Function)	The averaged psychometric function derived from a group of participants
Tactor	A combination of a vibration actuator/motor and a stimulus tip designed to induce tactile stimuli
ITD (Inter-Tactor Distance)	The center-to-center spacing between tactors in a tactile display

References

1. Elsayed, H. *et al.* VibroMap. *Proc. ACM Interact. Mob. Wearable Ubiquitous Technol.* **4,** 1–16 (2020).
2. Stronks, H. C., Parker, D. J. & Barnes, N. Vibrotactile Spatial Acuity and Intensity Discrimination on the Lower Back Using Coin Motors. *IEEE transactions on haptics* **9,** 446–454 (2016).
3. Weber, E. H., Helen, E. R. & David, J. M. *E.H. Weber on the Tactile Senses (translated from Original 1834)* (Psychology Press, 1834/2018).
4. Weinstein, S. Intensive and extensive aspects of tactile sensitivity as a function of body part, sex, and laterality. *The Skin Senses,* 195–222 (1968).
5. Gibson, G. O. & Craig, J. C. Tactile spatial sensitivity and anisotropy. *Perception & psychophysics* **67,** 1061–1079; 10.3758/bf03193632 (2005).
6. Mancini, F. *et al.* Whole-body mapping of spatial acuity for pain and touch. *Annals of neurology* **75,** 917–924 (2014).
7. Tong, J., Mao, O. & Goldreich, D. Two-point orientation discrimination versus the traditional two-point test for tactile spatial acuity assessment. *Frontiers in human neuroscience* **7,** 579; 10.3389/fnhum.2013.00579 (2013).
8. Eagleman, D. M. & Perrotta, M. V. The future of sensory substitution, addition, and expansion via haptic devices. *Front. Hum. Neurosci.* **16**; 10.3389/fnhum.2022.1055546 (2023).
9. Mayerhofer, A., Kirsch, J., Aust, G., Mense, S. & Engele, J. *Duale Reihe Anatomie.* 6th ed. (Thieme, Stuttgart, 2024).
